# Audit of accuracy of ultrasound-guided axillary core biopsy

**DOI:** 10.1186/bcr2974

**Published:** 2011-11-04

**Authors:** A Anand, R Henderson

**Affiliations:** 1 Darlington Memorial Hospital, Darlington, UK

## Introduction

Sentinel lymph node biopsy (SLNB) is the preferred method of axillary staging in breast cancer. If metastases are detected, axillary node clearance (ANC) is necessary. Preoperative detection of nodal metastases using ultrasound-guided core biopsy (USCB) allows the surgeon to proceed directly to ANC. Negative CBs do not exclude metastases. All patients still need SLNB. However, by minimising false negatives, unnecessary SLNBs can be minimised. We compared our USCB results with the results of subsequent SLNB + axillary node sampling (ANS) or ANC to assess our accuracy.

## Methods

We performed a retrospective audit of patients having USCB in primary breast cancer. We included patients presenting to one consultant radiologist firm from our unit's symptomatic breast clinic between 27 March 2007 and 7 December 2010. Our criteria for CB included cortical thickness >2 mm, loss of fatty hilum and longitudinal axis/transverse axis <2. We used a 14G Achieve needle to make four passes into the node.

## Results

Out of 41 CBs, on histology, eight were negative and 33 were positive. All positives were proven to be true positive at ANC. Seven out of eight negatives were found to be true negative (87.5% true negative) at SNB and ANS. One out of eight negative CBs was found to be false negative (12.5% false negative) requiring axillary clearance.

## Conclusion

We found that our results were comparable with published recent studies from the UK and abroad. Aggressive and more focused sampling could be suggested to further reduce false negatives.

